# Myeloid Diagnostic and Prognostic Markers of Immune Suppression in the Blood of Glioma Patients

**DOI:** 10.3389/fimmu.2021.809826

**Published:** 2022-01-07

**Authors:** Paola Del Bianco, Laura Pinton, Sara Magri, Stefania Canè, Elena Masetto, Daniela Basso, Marta Padovan, Francesco Volpin, Domenico d’Avella, Giuseppe Lombardi, Vittorina Zagonel, Vincenzo Bronte, Alessandro Della Puppa, Susanna Mandruzzato

**Affiliations:** ^1^ Department of Oncology, Oncology 1, Veneto Institute of Oncology IOV-IRCCS, Padova, Italy; ^2^ Department of Surgery, Oncology and Gastroenterology, University of Padova, Padova, Italy; ^3^ University Hospital and Department of Medicine, Verona, Italy; ^4^ Department of Medicine, University of Padova, Padova, Italy; ^5^ University Hospital of Padova, Neurosurgery Department, Padova, Italy; ^6^ Neurosurgery, Department of NEUROFARBA, Careggi University Hospital, University of Florence, Florence, Italy

**Keywords:** glioma, biomarkers, STAT3, myeloid-derived suppressor cell, arginase 1 (ARG1)

## Abstract

**Background:**

Although gliomas are confined to the central nervous system, their negative influence over the immune system extends to peripheral circulation. The immune suppression exerted by myeloid cells can affect both response to therapy and disease outcome. We analyzed the expansion of several myeloid parameters in the blood of low- and high-grade gliomas and assessed their relevance as biomarkers of disease and clinical outcome.

**Methods:**

Peripheral blood was obtained from 134 low- and high-grade glioma patients. CD14^+^, CD14^+^/p-STAT3^+^, CD14^+^/PD-L1^+^, CD15^+^ cells and four myeloid-derived suppressor cell (MDSC) subsets, were evaluated by flow cytometry. Arginase-1 (ARG1) quantity and activity was determined in the plasma. Multivariable logistic regression model was used to obtain a diagnostic score to discriminate glioma patients from healthy controls and between each glioma grade. A glioblastoma prognostic model was determined by multiple Cox regression using clinical and myeloid parameters.

**Results:**

Changes in myeloid parameters associated with immune suppression allowed to define a diagnostic score calculating the risk of being a glioma patient. The same parameters, together with age, permit to calculate the risk score in differentiating each glioma grade. A prognostic model for glioblastoma patients stemmed out from a Cox multiple analysis, highlighting the role of MDSC, p-STAT3, and ARG1 activity together with clinical parameters in predicting patient’s outcome.

**Conclusions:**

This work emphasizes the role of systemic immune suppression carried out by myeloid cells in gliomas. The identification of biomarkers associated with immune landscape, diagnosis, and outcome of glioblastoma patients lays the ground for their clinical use.

## Introduction

The studies on the relationship between immune system and gliomas revealed a strong control of the tumor on the existing antitumor activity, mainly due to a profound local and systemic immune suppression (1). One of these mechanisms depends on the release of soluble factors that drive the generation and recruitment of altered myeloid cells displaying a potent immune suppressive activity (2). Indeed, during tumor progression, myelopoiesis is diverted from its normal pathway and often results in the expansion and accumulation of myeloid-derived suppressor cells (MDSCs), a heterogeneous population frequently expanded in different types of cancer, impairing antitumor innate and adaptive immune responses (3, 4). The phenotype of MDSCs shows a complex plasticity that depends upon the particular combination of tumor-derived soluble factors that are present in the tumor microenvironment. In humans, three main subsets can be distinguished: polymorphonuclear MDSCs (PMN-MDSCs), monocytic MDSCs (M-MDSCs), and early-stage MDSCs (e-MDSCs) (4, 5), although each contains more than one cell population (6, 7). In line with their function of suppressing the immune response, MDSC levels correlate proportionally with tumor burden (8) and are associated with tumor progression and lack of response to therapy (9, 10). Concerning glioblastoma (glioma grade IV, GBM), previous works reported the expansion of subsets of MDSCs (11–15) and an association of levels of CD11^+^/CD33^+^/HLA-DR^low/−^ MDSCs with survival (16). Interestingly, based on these observations, a phase 0/I dose-escalation trial has been conducted in recurrent GBM with metronomic capecitabine, to reduce MDSC levels (17).

Among the mechanisms of the immune-suppressive machinery that have been described in MDSCs, the expression of programmed death-ligand 1 (PD-L1) and the activation of signal transducer and activator of transcription 3 (STAT-3) and of the enzyme arginase-1 (ARG1) have been reported (18–20). In fact, another mechanism by which immune cells promote cancer growth is the depletion of essential nutrients that are required by lymphocytes, like arginine. Reduction in arginine levels can be obtained by ARG1, resulting in antigen-activated T-cells proliferation arrest (21). High levels of arginase have been reported in several cancer types, thus providing an attractive target for anticancer immunotherapy (22). A few studies document the role of ARG1 (11, 23) and of PD-L1 (24) in the immune suppression in GBM, but data are scarce in circulating myeloid cells from low-grade gliomas (11).

Recently, suppressive M-MDSCs from pancreatic ductal carcinoma patients were characterized as STAT3^+^/ARG1^+^/CD14^+^ cells with a distinct gene signature in which STAT3 has a main role in driving MDSC function (19). Once activated, this pathway inhibits T-cell proliferation and reduces T-cell effector functions. The expansion of MDSCs can be considered not only as a hallmark of immune suppression but also as a biomarker of disease, or disease progression, and in fact, levels of circulating MDSCs have been regarded as a tool to monitor disease progression (25).

Biomarkers for patient stratification and for response to therapy are an important tool in oncology. In brain tumors, the possibility of using body fluids as a source of biomarkers to diagnose and define disease progression is attractive, since it is minimally invasive, thus circumventing the need of intracranial sampling. For this reason, circulating tumor cells, exosomes, proteins, nucleic acids, and metabolites have been proposed as potential biomarkers in gliomas, but, at present, none is yet in clinical use (26).

In this prospective study, we analyzed in the blood of healthy donors (HD) and of low- and high-grade glioma patients a number of soluble and cell-associated markers of myeloid cells to identify new biomarkers capable of predicting diagnosis and clinical outcome. The analysis of these myeloid parameters allowed to develop a diagnostic score that classified HDs versus glioma patients, and versus grade II, III, and IV glioma (GII, GIII, GIV) patients. Finally, a GIV prognostic risk model was identified that combines clinical and myeloid parameters.

## Methods

### Patients and Samples

**Figure 1:**
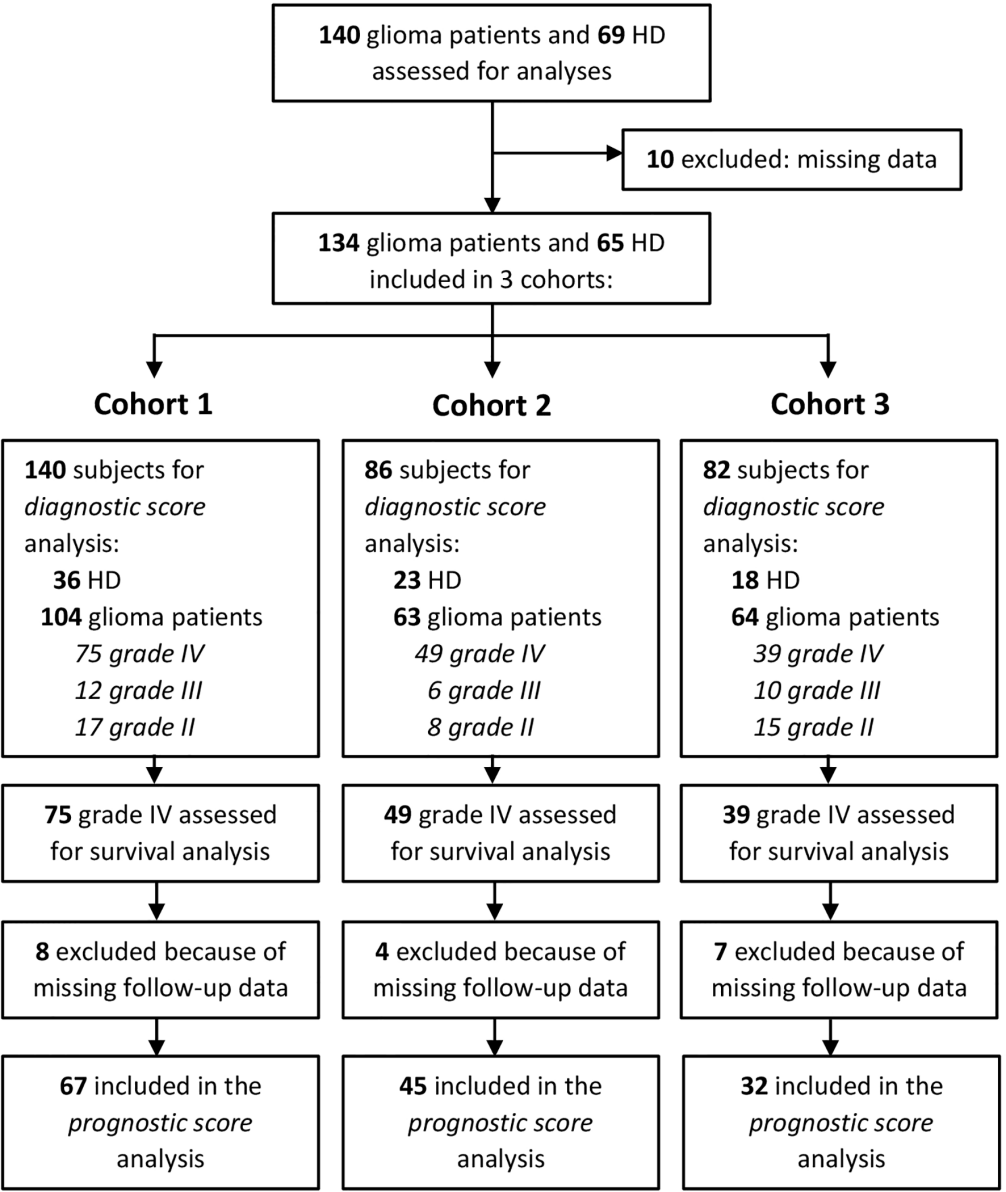
Consort statement.

Patients were prospectively recruited at the Department of Neurosurgery, Padova University Hospital, Italy, from 2016 to 2019, then followed at the Veneto Institute of Oncology, Padova, Italy. Patients were eligible if they were older than 18 years with histologically confirmed diagnosis of glioma. Clinical data and molecular analyses such as MGMT methylation status and IDH mutational status were also collected, prospectively. A total of 134 treatment-naive glioma patients (n = 19 grade II, n = 14 grade III, n = 101 grade IV, **Table 1**) undergoing surgery and 65 healthy donors (HDs), matched for age and sex, as control, were included in this study. Of these, 140 (104 patients and 36 HDs) were analyzed for myeloid-cell-associated markers (CD14^+^, CD15^+^, MDSC1, MDSC2, MDSC3, MDSC4; cohort 1), 86 (63 patients and 23 HDs) were analyzed for STAT3 and PD-L1 (cohort 2), and 82 (64 patients and 18 HDs) for ARG1 quantity and activity (cohort 3), as shown in **Supplementary Table S1**. Cohorts 1 and 2 shared 4 HDs and 15 patients; cohorts 1 and 3 shared 8 HDs and 30 patients; 26 patients were included in all 3 cohorts. Details of subject inclusion are reported in **Figure 1**. Only subjects with complete data were eligible for the analyses. The study protocol was reviewed and approved by the ethical committee of the IOV-IRCCS and of Padova University Hospital, and all patients gave their informed consent.

**Table 1:** Participant characteristics.

	Participant Characteristics				
	HD	Glioma Patients	Cohort 1	Cohort 2	Cohort 3
Total number	65	134 ➪	104	63	64
**Sex**					
Male (n)	35	81	64	36	40
Female (n)	30	53	40	27	24
**Median age**	55	59.5	59	63	56
Range	26–84	18–80	18–80	18–79	24–79
**WHO classification**					
WHO grade IV	NA	101	75	49	39
WHO grade III	NA	14	12	6	10
WHO grade II	NA	19	17	8	15
**IDH status**					
WT	NA	105	80	52	40
Mutated	NA	29	24	11	24
**MGMT status**					
Methylated	NA	63	49	31	29
Not methylated	NA	49	36	21	19
NA	NA	22	19	11	16

### Flow Cytometry

Peripheral blood was obtained from patients at surgery before anesthesia induction, or the day before surgery, and processed as previously described (27). To analyze myeloid cell subsets, 50 µl of fresh unrefrigerated whole blood from patients and from HDs were washed with phosphate-buffered saline (PBS) plus 1% fetal bovine serum (FBS, Gibco, Thermo Fisher Scientific, Waltham, MA, USA) and subsequently incubated with Fc-receptor blocking reagent (Miltenyi Biotec, Bergisch Gladbach, Germany) at 4°C for 10 min. Afterwards, cells were stained with mAbs, and at the end of incubation, cells were washed with PBS plus 1% FBS and centrifuged at 1,300 rpm for 6 min at 4°C. Red blood cells were lysed using Cal-Lyse whole blood lysing solution (Life Technologies, Thermo Fisher Scientific) according to manufacturer’s instructions and as previously described (6). MDSC subsets were identified by a seven-color staining, containing anti-CD11b Alexa700 (BD Biosciences, Becton Dickinson, Franklin Lakes, NJ, USA), anti-CD14 APC-H7 (BD Biosciences), anti-CD15 V450 (BD Biosciences), anti-CD33 PE-Cy7 (eBioscience, Thermo Fisher Scientific), anti-IL4Rα PE (R&D SYSTEMS, Minneapolis, MN, USA), Lineage cocktail (Lin) FITC (BD Biosciences), and anti-HLA-DR APC (BD Biosciences), and the immunophenotyping was standardized as described in **Supplementary Material**. Anti-CD274 (PD-L1) PE (eBioscience), anti-CD14 APC-H7 (BD Biosciences), and anti-HLA-DR APC (BD Biosciences) were used to analyze the expression of PD-L1 in myeloid cells. Fluorescence minus one (FMO) for HLA-DR, IL4Rα, and PD-L1 were used as negative controls.

To detect intranuclear p-STAT3, PBMCs were stained with anti-CD14 FITC (BD Biosciences), then fixed with 1% formaldehyde for 10 min at 37°C, washed twice with PBS 4% FBS and permeabilized with ice-cold 96% methanol for 15 min at −20°C. After removing the methanol by washing twice with PBS 4% FBS, PBMCs were stained with antihuman p-STAT3 (Tyr705) mAb (Cell Signaling Technology, Danvers, MA, USA) and then with donkey anti-rabbit AF-647 IgG antibody (BioLegend, San Diego, CA, USA) for 30 min at room temperature (RT). As positive control for p-STAT3 staining, a homogeneous batch of HepG2 cell line was used, treated with 80 ng/ml rhIL-6 (Thermo Fisher Scientific) for 10 min to induce STAT3 phosphorylation, and then, cells were fixed, permeabilized, and frozen. Single aliquots of HepG2 were thawed, and p-STAT3 staining was run in parallel to patient’s samples to check antibody performance. Data were acquired using a LSRII flow cytometer (BD Biosciences) equipped with four lasers (405, 488, 561, and 640 nm), and analysis was performed by FlowJo software v 7.6.5 (Becton Dickinson, Franklin Lakes, NJ, USA).

To analyze the intracellular expression of ARG1, PBMCs and PMNs were isolated from the peripheral blood of HDs and glioma patients as previously described (27), stained with anti-CD14 FITC (BD Biosciences) and anti-CD15 V450 (BD Biosciences); then, cells were fixed and permeabilized with Cytofix/Cytoperm (BD Biosciences) for 20 min at 4°C. Then, PBMCs and PMNs were stained for 30 min at 4°C with mouse monoclonal antibody anti-human ARG1 [clone 1.10 (9),] conjugated to Alexa Fluor (AF)-647 by SAIVI™ Rapid Antibody Labeling Kits (Thermo Fisher Scientific), following the manufacturer’s instructions. FMO for ARG1 was used as negative control. To acquire cells stained with ARG1, a FACSCelesta flow cytometer (BD Biosciences) was used, equipped with three lasers (405, 488, and 640 nm), and analysis was performed by FlowJo software v 7.6.5. All antibodies used for flow cytometry were titrated in a lot-dependent manner.

### Standardization of MDSCs Staining Acquisition and Analysis

To standardize MDSC subsets’ evaluation, a dilution of antibodies that maximizes the signal-to-noise ratio was chosen based on single antibodies titration. In addition, a protocol to monitor the performance of antibodies against HLA-DR and IL4Rα was set up. Briefly, an EBV-B cell line that constitutively expresses these markers at high expression intensity was used as reference, as previously described.^2^ To reduce inter-assay variance, the B-cell line was fixed and permeabilized in large batches, and a single vial was run in parallel to blood staining for each patient. Acquisition of control cells was performed before the blood sample to evaluate whether the mean fluorescence intensity (MFI) of HLA-DR or IL4Rα fell in the range of tolerance built by repeated staining of the control cells. In addition, for each patient, fluorescence minus one (FMO) for HLA-DR and IL4Rα were used as negative controls. To monitor the performance of the LSRII flow cytometer (BD Biosciences), the potential variation of the performance of the instrument was assessed, using a protocol after Perfetto et al. (28)

### Confocal Microscopy

PBMCs and PMNs were seeded on coverslips for 2 h in 24-well plates and washed three times with PBS to eliminate non-adhering cells, fixed with 4% paraformaldehyde (Sigma-Aldrich, Merck KGaA, Darmstadt, Germany) for 10 min at RT, then blocked with PBS containing 0.05% Triton and 20% normal goat serum (NGS Vector Laboratories, Burlingame, CA, USA) for 2 h at RT. Cells were stained for CD14 and ARG1 using an anti-CD14 Cy3 (Bioss, Woburn, MA, USA) and anti-human arginase-1 clone 1.10 conjugated with AF-647 and incubated overnight at 4°C. DNA was visualized with 4′,6-diamidino-2-phenylindole (DAPI) (Sigma-Aldrich). Samples were analyzed under a laser scanning confocal microscope (Leica TCS SP5, Leica Microsystems, Wetzlar, Germany) equipped with four lasers (405 nm/argon-458, 476, 488, 494, 514 nm/561 nm/633 nm), and results were analyzed by Las X (Leica Microsystems).

### Determination of ARG1 Levels and Activity

The plasma level of ARG1 was analyzed using Arginase Liver-Type Human ELISA (BioVendor Laboratory Medicine Inc., Brno, Czech Republic) following the manufacturer’s instructions. Plasma was obtained upon centrifugation over Ficoll–Paque Plus (GE Healthcare-Amersham, NJ, USA) of peripheral blood. The supernatant was collected and further spun at 1,300 rpm at 4°C for 6 min and stored at −80°C. Samples were assayed in duplicates, and ARG1 concentration was extrapolated from the standard curve. Arginase activity was tested in plasma samples by measuring the production of urea, at pH 7.1 and 9.5, as detailed in **Supplementary Materials and Methods**.

### Statistical Analysis

Continuous variables were summarized using median and interquartile range and categorical variables as frequencies and percentages. A two-tailed Mann–Whitney test, adjusted for sex and age, followed by the Benjamini–Hochberg multiple testing correction, was used to address the pairwise comparisons of each biomarker distribution between HDs and low- and high-grade glioma patients. Multiple logistic and multinomial logistic regression models were estimated to develop a diagnostic score using each biomarker categorized according to high and low levels. The selection of variables was based on Akaike’s information criterion in order to reduce model complexity. Optimal cut points for each soluble and cell-associated marker of myeloid cells were selected using a criterion based on minimization of the most frequent error (29). The odds ratios (ORs) were reported with their 95% confidence interval (CI). A repeated (three repeats) fivefold cross‐validation was utilized for internal validation. Accuracy was calculated to assess the prediction error. Among grade IV glioma patients, clinical outcome was analyzed in terms of overall survival (OS), defined as the time from the date of surgery to death. Patients who did not develop an event during the study period were censored at the date of last observation. The median follow-up time was based on the reverse Kaplan–Meier estimator. The survival probabilities were estimated using the Kaplan–Meier method, and median survival was reported with a 95% CI calculated according to Brookmeyer and Crowley. The association of clinical characteristics and markers with overall survival was investigated in multiple Cox proportional hazards regression models. No deviation from the proportional hazard assumption was found by the test statistic of Grambsch and Therneau. Clinical prognostic factors incorporated in the model include age at surgery, sex, Eastern Cooperative Oncology Group (ECOG) performance status (ECOG PS) (0–1, 2–4), type of surgery (radical, other), whether patients received Stupp’s treatment, O[6]-methylguanine-DNA methyltransferase (MGMT) promoter methylation (present, absent), and isocitrate dehydrogenase (IDH) status [mutated, wild type (WT)]. Markers within each cohort were dichotomized with cut points corresponding to the most significant relation with the outcome, estimated by maximizing the discriminative ability of the Cox model. The best model was selected with the lower Akaike information criteria, and the concordance index (C-index) was used to evaluate the discrimination of the model. Bootstraps with 1,000 resamples were calculated to correct the C-index.

All statistical tests were two-sided, and a p < 0.05 was considered statistically significant. Statistical analyses were performed using the RStudio (RStudio: Integrated Development for R. RStudio Inc., Boston, MA, USA).

## Results

### Expansion of Suppressive Myeloid Cell Subsets in the Peripheral Blood of Glioma Patients

**Figure 2:**
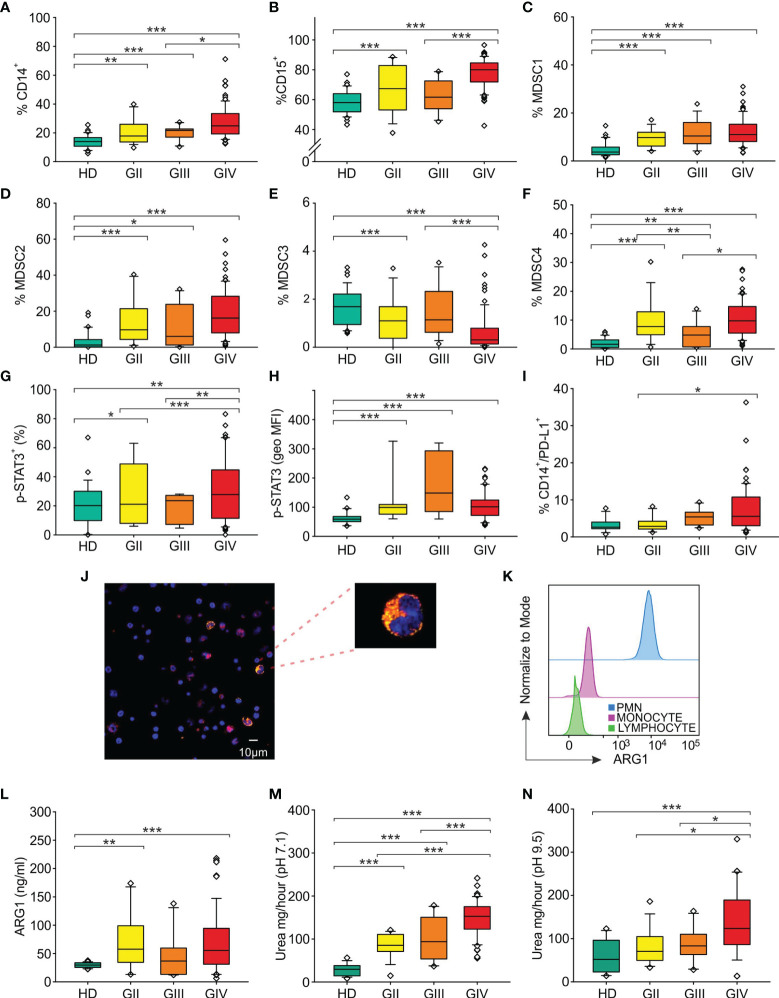
Distribution of circulating myeloid markers in glioma patients. Box plots show the median, 25th, and 75th percentile of the percentage of **(A)** monocytes (CD14^+^ cells among PBMCs) [36 HDs, 18 grade II gliomas (GII), 13 grade III gliomas (GIII) and 78 grade IV gliomas (GIV)], **(B)** granulocytes (CD15^+^ cells among peripheral blood leukocytes, PBLs) (36 HDs, 18 GII, 13 GIII, and 79 GIV), **(C)** MDSC1 (CD14^+^/IL4Rα^+^ cells among PBMCs) (36 HDs, 18 GII, 13 GIII, and 79 GIV), **(D)** MDSC2 (CD15^+^/IL4Rα^+^ cells among PMNs) (36 HDs, 17 GII, 12 GIII, and 79 GIV), **(E)** MDSC3 (Lin^−^/HLA-DR^−^/CD11b^+^/CD33^+^ cells) in CD15^−^ cells (36 HDs, 18 GII, 13 GIII, and 79 GIV), **(F)** MDSC4 (CD14^+^/HLA-DR^low/−^ cells among PBMCs) (36 HDs, 18 GII, 13 GIII, and 76 GIV), **(G)** p-STAT3^+^ in CD14^+^ cells among PBMCs (23 HDs, 8 GII, 6 GIII, and 50 GIV). **(H)** The geometric mean fluorescence intensity of p-STAT3 (p-STAT3, geo MFI) expression is shown compared to a negative control by flow cytometry in CD14^+^ cells among PBMCs of 23 HDs and glioma patients (8 GII, 6 GIII, and 50 GIV), following intracellular staining. **(I)** Surface expression of PD-L1 was evaluated in HDs and glioma patients, by gating CD14^+^/PD-L1^+^ cells among PBMCs (29 HDs, 14 GII, 10 GIII, and 61 GIV). Evaluation of blood ARG1 presence and activity. **(J)** Representative image of confocal microscopy analysis performed on PBMCs from glioma patients. ARG1^+^ cells are shown in yellow and CD14^+^ cells in red. Slides were analyzed at a 63× magnification, and cell size is reported by scale bar (10 µm). Upper right panel shows a 189× magnification of a monocyte positive for both ARG1 and CD14 markers from a GBM patient. **(K)** Flow cytometry analysis of ARG1 in PMNs (blue), monocytes (pink), and lymphocytes (green). **(L)** Levels of ARG1 evaluated by ELISA (22 HDs, 15 GII, 10 GIII, and 57 GIV). ARG1 functional activity was tested by urea assay in the plasma of HDs and glioma patients at a 7.1 pH (18 HDs, 15 GII, 10 GIII, and 57 GIV) **(M)** and at pH 9.5 pH (18 HDs, 15 GII, 10 GIII, and 39 GIV) **(N)**. Whiskers extend to 1.5 interquartile range, and outliers are shown by dots. Only statistically significant comparisons are reported in the figure: ***<0.001, **<0.01, and *<0.05.

To determine the role of circulating immune parameters related to immune suppression in the clinical outcome of glioma patients, we set out to perform an immunophenotypical analysis of circulating myeloid cells by multicolor flow cytometry in 134 patients undergoing surgery for a suspect glioma. To this aim peripheral blood samples were prospectively collected from three cohorts of patients and control HDs, as detailed in **Figure 1**, and parameters analyzed in each of the three cohorts are indicated in **Supplementary Table S1**. As previously reported by us and others (30–32), grade IV patients had a significant increase in the percentage of CD14^+^-circulating monocytes as compared to age- and sex-matched HDs, and a significant increase in PMNs, defined as CD15^+^ cells (**Figures 2A, B**). When we analyzed lower grade gliomas, we observed that also these patients were characterized by a significant expansion of both monocytes and PMNs, with a significant increase going from lower grade to grade IV [**Figure 2A** (for monocytes) and **2B** (PMN)]. Although some of these glioma patients underwent preoperative dexamethasone and it is known that this treatment increases PMNs (33, 34), no clear data exist regarding its effect on monocytes (35). However, we did not find a significant difference in CD14^+^ cells from patients with or without preoperative dexamethasone (**Supplementary Figure S1**). Collectively, these results indicate that the increase in CD14^+^ monocytes in glioma patients is not due to dexamethasone and suggest a dysregulation of the myeloid compartment. In the same blood samples, we also evaluated MDSC levels. Previous studies analyzed MDSCs in glioma patients, but few of them analyzed more than one MDSC subset at a time, and most studies evaluated MDSCs among PBMCs, thus excluding the potential presence of PMN-MDSC subsets present in whole blood, discarded after a Ficoll gradient. In addition, also cryopreservation is well known to influence the evaluation of PMN-MDSCs. Based on these considerations, we used a standardized seven-color panel, stemming from our previous experience (6, 7, 36), to detect in fresh whole blood the simultaneous presence of M-MDSC1 (CD14^+^/IL4Rα^+^), PMN-MDSC2 (CD15^+^/IL4Rα^+^), e-MDSC3 (Lin^−^/HLA-DR^−^/CD11b^+^/CD33^+^) and M-MDSC4 (CD14^+^/HLA-DR^low/−^). All glioma patients had a significant expansion of circulating MDSC1, 2, and 4 subsets in comparison to age-matched HDs (**Figures 2C, D, F**). On the contrary, MDSC3 levels were significantly decreased in glioma grade II and IV patients compared to HDs and in grade IV compared to grade III (**Figure 2E**). Interestingly, MDSC4 were significantly increased in grade IV compared to grade III gliomas, raising the possibility to use this marker in longitudinal studies to monitor evolution of grade III gliomas into higher grade (**Figure 2F**), since it is well known that GIII gliomas eventually evolve to grade IV.

### Activation of STAT3/PD-L1 Axis in Circulating Monocytes From Glioma Patients

We next investigated in circulating monocytes the activation of STAT3, one of the key players regulating tolerogenic activities of tumor-associated myeloid cells (20), by analyzing p-STAT3 expression in CD14^+^ cells and found that its intensity significantly increased in all glioma grade compared to HD (**Figure 2H**), thus suggesting an active involvement of this transcription factor in the modulation of immune suppression.

Once phosphorylated, STAT3 moves to the nucleus, where it can induce the expression of PD-L1 by binding to its promoter and activating its transcription (37). Thus, to further analyze the regulation carried out by STAT3, we evaluated the expression of PD-L1 on circulating monocytes and found that the percentage of monocytes expressing PD-L1 was significantly increased on grade III and IV gliomas, but not in grade II (**Figure 2I**).

All together, these observations indicate that increased glioma grading is associated with a rise in CD14^+^ cells expressing activated STAT3 and PD-L1, suggesting their potential use as blood biomarkers.

### ARG1 Activity Increases With Tumor Stage

ARG1, an enzyme constitutively expressed in PMNs and stored within intracellular granules, is a downstream target of activated STAT3, and in circulating MDSCs from cancer patients, STAT3 controls the immune suppressive activity (20). We thus investigated the presence of ARG1 in circulating PBMCs from glioma patients, by using confocal microscopy (**Figure 2J**) and flow cytometry analysis (**Figure 2K**). With both techniques, we observed the presence of a fraction of CD14^+^ monocytes expressing ARG1 (18.8% by flow cytometry analysis and 21.4% by confocal microscopy) localized in the cytoplasm of the cells.

Previously, elevated circulating levels of ARG1 in GBM patients have been associated with PMN degranulation and immunosuppression (23). In addition, we demonstrated that high serum levels of ARG1 in pancreatic ductal adenocarcinoma patients are associated with high ARG1 activity (19). We thus measured ARG1 levels in the plasma samples obtained from 64 glioma patients (15 GII, 10 GIII and 39 GIV) and found that ARG1 levels in glioma patients were significantly higher compared to HD control (**Figure 2L**). We then assessed ARG1 enzymatic activity at both pH 7.1 and pH 9.5 (**Figures 2M, N**). In both conditions, serum from glioma patients showed a significant increase in ARG1 activity that peaked in grade IV gliomas. Interestingly, ARG1 activity evaluated at pH 7.1 positively correlated with tumor grade, increasing from a median activity of 29.6 mg/h of urea in HD to 85.4 mg/h in GII, 93.9 and 152.8 mg/h in GIII and GIV, arguing for ARG1 activity as a potential marker of glioma progression from grade III to GIV, although longitudinal studies are required to confirm and strengthen this conclusion.

### Development of a Diagnostic Score to Identify Biomarkers Associated With Disease and With Disease Stage

To define a diagnostic score, each biomarker was categorized according to high and low levels, and the association with the disease was tested first by univariate analysis (**Supplementary Tables 2, 3**), followed by multiple logistic regression models considering in the stepwise model selection all markers within each cohort, together with age and sex. Analysis was performed including glioma patients as a single group, in a case–control study (univariate in **Supplementary Table S2** and multivariate in **Table 2**), or divided on the basis of pathological stage, from grade II to IV (univariate in **Supplementary Table S3** and multivariate in **Table 3**). When cell-associated myeloid markers were considered as biomarkers to differentiate HD from glioma patients, levels of CD15^+^ cells, MDSC1, and MDSC3 emerged as independent factors predicting the presence of disease (**Table 2**), with an overall accuracy of 87.1%. In detail, high levels of CD15^+^ cells and MDSC1 were significantly associated with a high risk of disease (adjusted OR, 7.2; 95% CI, 2.2–24.1 and adjusted OR, 40.1; 95% CI, 10.1–160.4, respectively), while high levels of MDSC3 showed a significantly lower probability to develop disease (adjusted OR, 0.1; 95% CI, 0.02–0.4). The logit transformation of the probability of glioma (any grade) risk was calculated as follows:


$$logit(p)=-1.372+1.976\times CD15^{+}+3.691\times MDSC1-2.413\times MDSC3$$

**Table 2:** Factors for glioma risk (multiple analysis with logistic regression model).

		E/N	Adjusted OR (95% CI)	*p* (LR test)
**Cohort 1**				
% CD15^+^	Low	16/39	1	
	High	88/101	7.2 (2.2–24.1)	<0.001
% MDSC1	Low	12/40	1	
	High	92/100	40.1 (10.0–160.4)	<0.001
% MDSC3	Low	89/105	1	
	High	15/35	0.1 (0.02–0.4)	<0.001
**Cohort 2**				
p-STAT3 (geo MFI)	Low	13/31	1	
	High	50/55	13.8 (4.3–44.3)	<0.001
**Cohort 3**				
Urea pH 7.1	Low	4/21	1	
	High	60/61	255 (26.71–2,434)	<0.001

OR, odds ratio; 95% CI, 95% confidence interval; E, number of glioma patients; N, total number of subjects; LR, likelihood ratio.

**Table 3:** Factors for glioma grade risk (multiple analysis with multinomial logistic regression model).

		Adjusted OR (95% CI)	Adjusted OR (95% CI)	Adjusted OR (95% CI)
		GIV	GIII	GII
**Cohort 1**				
Age	Cont.	1.01 (0.96–1.07)	0.95 (0.9–1.01)	0.9 (0.85–0.96)**
% CD15^+^	Low	1	1	1
	High	32.6 (6.3–168.3)***	2.9 (0.6–15.2)	2.8 (0.5–14.7)
% MDSC1	Low	1	1	1
	High	65.0 (12.5–338.2)***	28.0 (4.0–194.7)***	28.8 (4.3–193.7)***
% MDSC3	Low	1	1	1
	High	0.07 (0.01–0.35)**	0.19 (0.03–1.11)	0.06 (0.01–0.44)**
**Cohort 2**				
Age	Cont.	1.06(1.0–1.13)*	0.98(0.91–1.07)	0.92(0.85–1)
p-STAT3 (geo MFI)	Low	1	1	1
	High	11.2 (3.3–38.6)***	17.6 (1.65–188.5)*	17.3 (1.6–192)*
**Cohort 3**				
Age	Cont.	1.18 (1.06–1.32)**	1.09 (0.98–1.21)	1.04 (0.94–1.15)
Urea pH 7.1	Low	1	1	1
	High	8382 (124.4–564647)***	291.8 (7.74–11009.3)**	433.5 (12.6–14858)***

Signiﬁcance levels: ***<0.001, **<0.01, and *<0.05

Of note, when glioma patients were classified according to their grade, CD15^+^ cells, MDSC1, and MDSC3 remained independent significant predictors of GIV, and MDSC1 was the biomarker significantly associated to all grades of disease (**Table 3**, accuracy of 72.9%). In this case, the formula for the risk score was:


$$logit(p_{GIV})=-3.953+0.011\times age+3.484\times CD15^{+}+4.175\times MDSC1-2.630\times MDSC3$$

$$logit(p_{GII})=3.201-0.104\times age+1.018\times CD15^{+}+3.362\times MDSC1-2.749\times MDSC3$$

$$logit(p_{GIII})=0.208-0.052\times age+1.066\times CD15^{+}+3.333\times MDSC1-1.651\times MDSC3$$

Regarding PD-L1 and p-STAT3 expression, only the shift of intensity of expression of activated STAT-3 in monocytes (p-STAT3) remained an independent factor predicting the presence of disease (**Table 2**, cohort 2), with an overall accuracy of 79.1%. In detail, high levels of expression of p-STAT3 were significantly associated with a high risk of disease (adjusted OR, 13.8; 95% CI, 4.3–44.3), and the final score was *logit*(*p*) = –0.3254 + 2.628 × *p*-*STAT*3 (*geo MFI*).

When glioma patients were considered according to their grade, intensity of p-STAT3 was confirmed to be an independent significant predictor of all grades of disease (**Table 2**, accuracy of 68.6%). From these results, it thus appears that high levels of STAT3 activation in monocytes is not only a marker of immune suppression but also a biomarker of disease.

The same analysis performed with soluble biomarkers identified ARG1 activity at physiological pH (urea pH 7.1) as an independent risk factor of disease (adjusted OR, 255; 95% CI, 26.7–2434), with an overall accuracy of 94% and *logit*(*p*) = –1.447 + 5.541 × *Urea pH* 7.1.

When this analysis considered glioma patients according to their grade, ARG1 activity at pH 7.1 remained an independent significant predictor of all grades of disease and, interestingly, significantly discriminated HD from low-grade gliomas, thus indicating that it is an early biomarker of glioma disease (**Table 3**, cohort 3), with an accuracy of 72%.

### Development of a Grade IV Glioma Prognostic Model

We next evaluated the prognostic role of the myeloid-associated biomarkers present in this study to predict the outcome of grade IV glioma patients by performing a univariate analysis (**Supplementary Table 4**) and then using multiple survival analyses (**Table 4**). Both analyses examined a cohort of 67 patients for myeloid cell markers (cohort 1), of 45 patients for PD-L1 and p-STAT3 (cohort 2), and of 32 patients for ARG1 markers (cohort 3) with available clinical and follow-up data (**Supplementary Table S5**). At an estimated median follow-up time of 33.2 months (95% CI, 30.5–49.1) for cohort 1, 27.6 months (95% CI, 19.3–30.5) for cohort 2, and 33.2 months (95% CI, 28.9–37.7) for cohort 3, median OS times were 12.6 months (95% CI, 10.6–18), 12.5 months (95% CI, 6.2–16.7), and 12.0 months (95% CI, 6.5–28.5), respectively.

**Table 4:** Factors for grade IV glioma patients’ survival (multivariate analysis with Cox proportional hazards model).

		E/N	Median (95% CI)	Adjusted HR (95% CI)	*p* (LR test)
**Cohort 1**					
ECOG PS	0–1	32/41	18.3 (14.0,22.1)	1	
	2–3	24/26	6.0 (4.2,10.6)	2.8 (1.5,4.9)	<0.001
Surgery	Other	47/53	11.2 (6.4,14.4)	1	
	Radical	9/14	25.9 (12.0, NE)	0.4 (0.2,0.8)	0.009
MGMT promoter	No	28/30	11.3 (6.5,13.0)	1	
Methylation	Yes	28/37	18.0 (10.8,22.8)	0.5 (0.3,0.9)	0.015
% MDSC2	Low	14/20	21.4 (5.9, 33.0)	1	
	High	42/47	11.5 (7.7, 14.4)	1.8 (1.0,3.4)	0.047
**Cohort 2**					
Surgery	Other	31/39	10.6 (5.9,16.0)	1	
	Radical	1/6	–	0.1 (0.01,0.8)	0.002
Stupp’s regimen	No	8/9	2.7 (1.8,8.2)	1	
	Yes	24/36	15.5 (9.2,22.8)	0.2 (0.09,0.5)	0.002
MGMT promoter	No	16/18	8.5 (3.1,13.0)	1	
Methylation	Yes	16/27	21.6 (6.2, NE)	0.2 (0.1,0.5)	<0.001
p-STAT3 (geo MFI)	Low	22/30	15.4 (8.7, 21.6)	1	
	High	10/15	6.2 (2.7, NE)	4.4 (1.7,11.6)	0.003
**Cohort 3**					
Stupp’s regimen	No	5/5	2.3 (0.6–NE)	1	
	Yes	18/27	20.0 (8.2–33.0)	0.2 (0.05,0.5)	0.005
Urea pH 9.5	Low	7/16	–	1	
	High	16/16	8.0 (5.7–13.0)	3.7 (1.4,9.9)	0.007

HR, hazard ratio; 95% CI, 95% confidence interval; E, number of deaths; N, total number of glioblastoma patients; NE, not estimable; LR, likelihood ratio.

In the multiple Cox regression model for the myeloid cell-associated markers, elevated MDSC2 levels remained significantly associated with worse OS [hazard ratio (HR) =1.8; 95% CI, 1.0–3.4], in addition to ECOG PS, surgery, and MGMT methylation (**Table 4**; C-index = 0.74). The prognostic index derived from the model was:


Prognostic risk score=1.017×PS−0.935×Surgery−0.689×MGMT+0.607×MDSC2

In light of the recent results of sex differences in MDSC levels in GBM patients (38), we analyzed MDSC1-4 levels according to sex and found a significant effect of MDSC3 in male and not in female patients (**Supplementary Table 6**), but this difference was not retained in the prognostic score.

Among STAT3 and PD-L1 markers, elevated levels of expression of p-STAT3 were significantly associated with worse overall survival (HR = 4.43; 95% CI, 1.7–11.6), in addition to surgery, Stupp and MGMT methylation (**Table 4**; C-index = 0.75), *Prognostic risk score* = –2.323 × *Surgery* – 1.551 × *Stupp* – 1.691 × *MGMT* + 1.488 × p-*STAT*3 (*geo MFI*).

The multiple prognostic model for ARG1 identified high levels of ARG1 activity at pH 9.5 as a risk factor for survival (HR = 3.7; 95% CI, 1.4–9.9), in addition to Stupp treatment (**Table 4**; C-index = 0.76),


Prognostic risk score=−1.838×Stupp+1.317×Urea pH 9.5

## Discussion

In the present study, we evaluated the role of a number of factors that have been previously associated with immune suppressive activity due to myeloid cells in different tumor types. For example, in several studies, a higher pretreatment MDSC level was significantly associated with worse OS (3, 39). Our study evaluated four subsets of MDSCs from low- to high-grade gliomas and found that three out of the four subsets are significantly increased in patients with increasing disease stage, thus reinforcing the notion that MDSCs are associated with disease progression, as previously reported (11, 16). In addition, we found that levels of M-MDSC1 are also an independent factor significantly associated with a high risk of disease in a case–control study and that it remained an independent factor discriminating different pathological stages of gliomas (**Table 2**). Thus, determination of MDSC1 levels represents a valuable tool associated with glioma diagnosis. Determination of this parameter was carried out by multiparametric flow cytometry, and we previously showed that monitoring MDSC subsets poses the problem of a lack of harmonization across different laboratories and that standardization of common parameters is a necessary step to obtain Intra- and inter-laboratory reproducibility results (7). Accordingly, for this study, we standardized reagents and instrument for all the samples acquired to guarantee a reliable comparison of the data across an extended period of time. As far as it concerns the prognostic role of MDSCs, we found that another subset of MDSCs was significantly associated with outcome, since high levels of PMN-MDSC2 before surgical resection were associated with worse OS. On the other side, immature e-MDSC3 levels were significantly reduced in GII and GIV, as observed in a previous study with melanoma patients (6), and a prognostic independent risk factor for glioma versus HDs and for discriminating different glioma pathological stages (**Table 2**). Reduction in this immature myeloid subset could be sustained by a dynamic but altered process of myeloid differentiation in cancer patients, giving rise to more differentiated myeloid cells toward monocytic or granulocytic MDSCs that, in fact, are increased in these patients. In line with this hypothesis, MDSC3 could represent a pool of circulating immature cells that gives rise to other MDSC subsets. Blood MDSC determination is a feasible option, while tracking MDSCs in the tumor specimen is a challenging task, since such cells are virtually indistinguishable from tissue macrophages, as they share myeloid markers and the functional immune-suppressive activity. In addition, it has been demonstrated that MDSCs differentiate to tumor-associated macrophages in the tumor microenvironment (40, 41).

Another finding that highlights an altered myelopoiesis in GBM patients is the increased presence of monocytes and PMNs, beside MDSC1, 2, and 4 (**Figure 2**). Accordingly, expanded monocytes bear markers of immune suppression, such as p-STAT3, and of immune dysfunction, like PD-L1, highlighting their involvement in the immune derangement process in gliomas. Furthermore, the intensity of p-STAT3 in monocytes is another independent prognostic factor capable to discriminate gliomas versus HDs. As previously reported, STAT3 regulates ARG1 in MDSCs from cancer patients (20), and in our study, we found that its quantity and activity are significantly increased in the blood of glioma patients, in line with a previous work (11). In addition, its activity at pH 7.1 is another independent prognostic factor with diagnostic potential, but its activity at pH 9.5 is a prognostic factor for survival, independent of the treatment. Future studies will have to address the source and the role of this enzyme in the blood of glioma patients. In particular, secreted ARG1 is active as a full-length protein at alkaline pH, while it is inactive at neutral pH unless cleaved by PMN-derived proteases (42). Thus, it will be interesting to understand whether ARG1 is actively secreted from the granules of PMNs, or released from immune-suppressive monocytes, or M-MDSC, and whether an activation step is required to fully activate its potential. In this respect, recent results from our laboratories indicate that this enzyme has a complex and important role in immune suppression in pancreatic ductal adenocarcinoma and that exploitation of this pathway may enhance cancer immunotherapy (S. Canè, submitted manuscript), thus raising the possibility of exploiting its use also in GBM.

All the results presented in this work require a validation in future independent studies. Of note, arginase activity at neutral pH and intensity of activated STAT3 hold potential to discriminate between the different glioma grading and could also be used to monitor in glioma patients transition from low to high grade. Finally, monitoring the level of these biomarkers during treatment could be useful to link their changes with clinical outcome, especially in the context of new immunotherapeutic approaches. To date, immune-checkpoint inhibitors did not improve survival in glioma patients (43, 44). The identification of new prognostic factors like STAT3 and ARG1 might represent a critical step toward the development of new successful strategies of intervention in GBM. In fact, drugs targeting these molecular factors could be associated to immune-checkpoint inhibitor in order to increase their efficacy in glioma patients. In conclusion, results from this study indicate that dysfunctional myeloid cells and soluble factors in glioma patients not only may be a potential source of circulating biomarkers associated with disease stage and clinical outcome but also highlight the altered interplay between immune system and tumor.

## Data Availability Statement

The raw data supporting the conclusions of this article will be made available by the authors, upon reasonable request.

## Ethics Statement

The studies involving human participants were reviewed and approved by Veneto Institute of Oncology IRCCS of Padova, Italy (MDSC_SNC 2016/13) and of Padova University Hospital (MDSC/SNC, 3848/AO/16). The patients/participants provided their written informed consent to participate in this study.

## Author Contributions

PDB: conceptualization, methodology, validation, formal analysis, and writing. LP: investigation, data curation, analysis, and writing. SaM: investigation, analysis, and writing. SC: investigation and analysis. EM: investigation and analysis. DB: resources. MP: resources and data curation. FV: resources. DA: resources. GL: resources and data curation. VZ: resources. VB: resources. ADP: conceptualization and resources. SuM: conceptualization, methodology, resources, writing, supervision, project administration, and funding acquisition.

## Funding

This work was supported by TRANSCAN-2, ERA-NET to SuM, Padova University, DiSCOG Department, grants number BIRD205873/20 and BIRD188051/18, Ministero della Salute (RF-2019-12369251 to SuM), and of IOV-IRCCS (BIOV19MANDR to SuM).

## Conflict of Interest

The authors declare that the research was conducted in the absence of any commercial or financial relationships that could be construed as a potential conflict of interest.

## Publisher’s Note

All claims expressed in this article are solely those of the authors and do not necessarily represent those of their affiliated organizations, or those of the publisher, the editors and the reviewers. Any product that may be evaluated in this article, or claim that may be made by its manufacturer, is not guaranteed or endorsed by the publisher.
