# Supplementary material for: Myeloid Diagnostic and Prognostic Markers of Immune Suppression in the Blood of Glioma Patients
Source: Front Immunol. 2022 Jan 7;12:809826. doi: 10.3389/fimmu.2021.809826 (PMC8777055; doi:10.3389/fimmu.2021.809826)
Supplement: Supplementary file 1 [file DataSheet_1.docx]

Supplementary Material

# Supplementary Materials and Methods

**MGMT methylation status and IDH mutational status analyses**

IDH1 and IDH2 mutational status were analyzed by Sanger sequencing. MGMT methylation status was performed by pyrosequencing and a cut-off of 7% was considered for assessing as unmethylated and methylated.

**Determination of ARG1 activity**

To eliminate residual cells potentially present in the plasma, samples were treated with 50 μl of lysis buffer (0.1% Triton X-100, 100 μg/ml pepstatin, 100 μg/ml aprotinin, and 100 μg/ml antipain) and shacked at 37°C for 30 minutes. Then, 60 μl of 25 mM Tris-HCl and 2 mM MnCl_2_ were added, and the solution was heated for 10 min at 56°C. The solution was hydrolyzed with 100 μl of 500 mM l-arginine dissolved in either carbonate buffer (pH 9.5) or phosphate buffer (pH 7.1), incubated at 37°C for 60–120 min and the reaction was stopped with 800 μl of H_2_SO_4_ (96%)/H_3_PO_4_ (85%)/H_2_O. The urea concentration was measured at 540 nM after addition of 40 μl of α-isonitrosopropiophenone (dissolved in 100% ethanol), followed by heating at 95°C for 15–40 min. Samples were assayed in duplicates and urea concentration was derived from a standard curve.

# Supplementary Figure and Tables


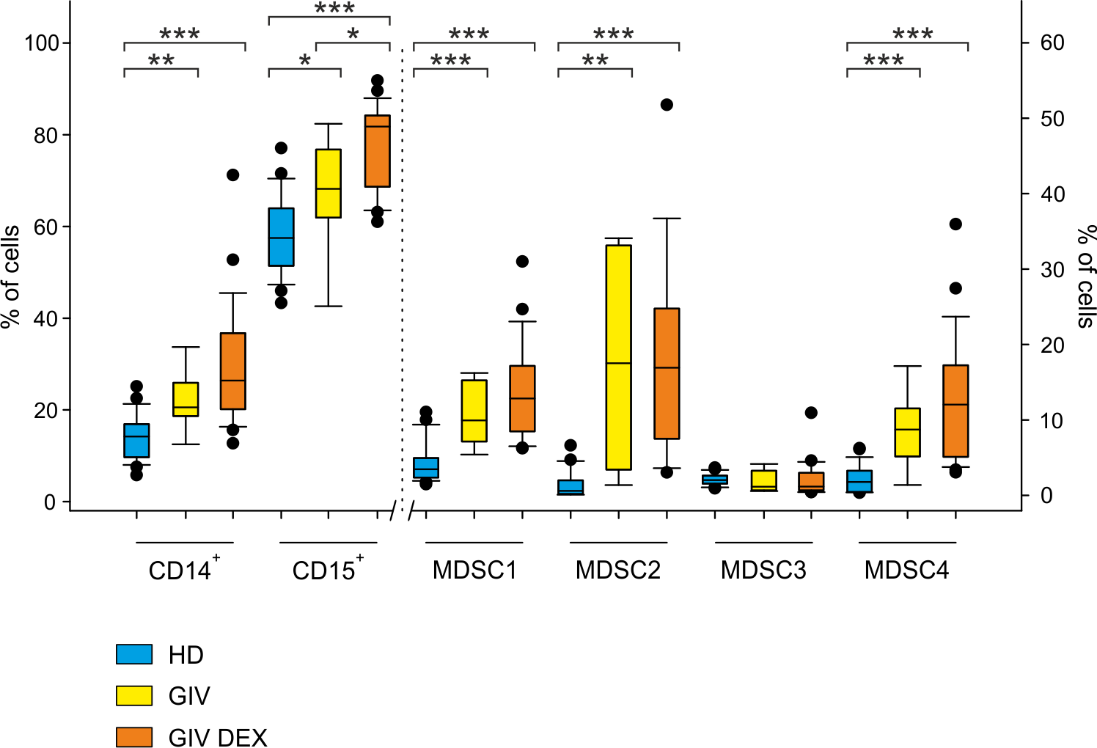


**Supplementary Figure 1.** Distribution of circulating myeloid cells in grade IV glioma (GIV) patients treated preoperatively with dexamethasone. Orange boxes indicate patients treated with dexamethasone (GIV DEX), yellow boxes refer to untreated patients (GIV). Box Plots show the median, 25th and 75th percentile of the percentage of monocytes (27 HDs, 8 GIV and 25 GIV DEX), granulocytes (26 HDs, 8 GIV and 25 GIV DEX), MDSC1, (27 HDs, 8 GIV and 25 GIV DEX), MDSC2 (27 HDs, 6 GIV and 19 GIV DEX), MDSC3 in CD15^-^ cells (27 HDs, 8 GIV and 22 GIV DEX), MDSC4 (27 HDs, 8 GIV and 24 GIV DEX). Whiskers extend to 1.5 inter-quartile range and outliers are shown by dots. Signiﬁcance levels evaluated by t-test: <0.001: ***; <0.01: **; <0.05: *.

**Supplementary Table 1.** Immunological variables analyzed in the study

| **Variable** | **Cohort** | **Determination** | **Source** |
| --- | --- | --- | --- |
| CD14^+^ cells | 1 | FCM, morphological gate (FSC/SSC) on PBMC | WB |
| CD15^+^ cells | 1 | FCM, morphological gate (FSC/SSC) on PBL | WB |
| MDSC1 | 1 | FCM, CD14^+^/CD124^+^ cells with morphological gate (FSC/SSC)  on PBMC | WB |
| MDSC2 | 1 | FCM, CD15^+^/CD124^+^ cells with morphological gate (FSC/SSC)  on PMN | WB |
| MDSC3 | 1 | FCM, Lin^-^/HLA-DR^-^/CD11b^+^/CD33^+^ gated on CD15^-^ cells among PBL | WB |
| MDSC4 | 1 | FCM, CD14^+^/HLA-DR^-/low^ with morphological gate (FSC/SSC)  on PBMC | WB |
| CD14^+^/PD-L1^+^ cells | 2 | FCM, morphological gate (FSC/SSC) on PBMC  followed by a gate on CD14^+^ cells | WB |
| p-STAT3 (%) | 2 | FCM, percentage of p-STAT3^+^ cells among CD14^+^ cells | PBMC |
| p-STAT3 (geo MFI) | 2 | FCM, geo MFI of p-STAT3 in CD14^+^ cells | PBMC |
| ARG1 | 3 | ELISA assay | Plasma |
| Urea pH 7.1 | 3 | ARG1 activity based on urea determination at pH 7.1 | Plasma |
| Urea pH 9.5 | 3 | ARG1 activity based on urea determination at pH 9.5 | Plasma |

**Abbreviations:** FCM flow cytometry, WB whole blood

**Supplementary Table 2.** Factors for glioma risk (univariate analysis with logistic regression model).

|  |  | **E/N** | **OR (95% CI)** | **p-value** |
| --- | --- | --- | --- | --- |
| **Cohort 1** |  |  |  |  |
| CD14^+^ | Low | 15/38 | Ref |  |
|  | High | 89/102 | 10.5 (4.4─25.1) | *<0.001* |
| CD15^+^ | Low | 16/39 | Ref |  |
|  | High | 88/101 | 9.7 (4.1─23.1) | *<0.001* |
| MDSC1 | Low | 12/40 | Ref |  |
|  | High | 92/100 | 26.8 (10.0─72.2) | *<0.001* |
| MDSC2 | Low | 13/39 | Ref |  |
|  | High | 91/101 | 18.2 (7.2─46.2) | *<0.001* |
| MDSC3 | Low | 89/105 | Ref |  |
|  | High | 15/35 | 0.1 (0.1─0.3) | *<0.001* |
| MDSC4 | Low | 13/36 | Ref |  |
|  | High | 91/104 | 12.4 (5.1─30.3) | *<0.001* |
| **Cohort 2** |  |  |  |  |
| CD14^+/^PD-L1^+^ | Low | 15/21 | Ref |  |
|  | High | 48/65 | 1.1 (0.4─3.3) | *0.83* |
| p-STAT3 (geo MFI) | Low | 13/31 | Ref |  |
|  | High | 50/55 | 13.8 (4.3─44.3) | *<0.001* |
| p-STAT3 (%) | Low | 19/23 | Ref |  |
|  | High | 44/63 | 0.5 (0.1─1.6) | *0.24* |
| **Cohort 3** |  |  |  |  |
| ARG1 | Low | 19/20 | Ref |  |
|  | High | 45/62 | 0.14 (0.02─1.12) | *0.064* |
| Urea pH 7.1 | Low | 4/21 | Ref |  |
|  | High | 60/61 | 255 (26.71─2434) | *<0.001* |
| Urea pH 9.5 | Low | 10/19 | Ref |  |
|  | High | 54/63 | 5.4 (1.72─16.95) | *0.004* |

**Abbreviations:** OR, odds ratio, 95% CI, 95% confidence interval; E, number of glioma patients; N, total number of subjects.

**Supplementary Table 3.** Factors for glioma grade risk (univariate analysis with multinomial logistic regression model).

|  |  | **OR (95% CI)** | **OR (95% CI)** | **OR (95% CI)** |
| --- | --- | --- | --- | --- |
|  |  | **GIV** | **GIII** | **GII** |
| **Cohort 1** |  |  |  |  |
| Age | Cont. | 1.02 (0.99─1.05) | 0.95 (0.91─1.0) | 0.91 (0.87─0.96)*** |
| CD14^+^ | Low | Ref | Ref | Ref |
|  | High | 17.19 (6.12─48.3)*** | 8.85 (1.68─46.69)* | 3.24 (0.97─10.82) |
| CD15^+^ | Low | Ref | Ref | Ref |
|  | High | 31.4 (9.32─105.88)*** | 2.48 (0.65─9.4) | 2.53 (0.78─8.24) |
| MDSC1 | Low | Ref | Ref | Ref |
|  | High | 34.0 (11.25─102.73)*** | 17.5 (3.17─96.7)** | 16.33 (3.74─71.31)*** |
| MDSC2 | Low | Ref | Ref | Ref |
|  | High | 25.26 (8.69─73.37)*** | 5.2 (1.28─21.18)* | 19.5 (3.76─101.1)*** |
| MDSC3 | Low | Ref | Ref | Ref |
|  | High | 0.1 (0.04─0.26)*** | 0.4 (0.1─1.57) | 0.17 (0.04─0.7)* |
| MDSC4 | Low | Ref | Ref | Ref |
|  | High | 20.35 (6.94─59.69)*** | 2.48 (0.65─9.4) | 13.27 (2.61─67.37)** |
| **Cohort 2** |  |  |  |  |
| Age | Cont. | 1.07 (1.02─1.12)** | 0.98 (0.91─1.06) | 0.91 (0.84─0.98)* |
| CD14^+^/PD-L1^+^ | Low | Ref | Ref | Ref |
|  | High | 1.22 (0.39─3.84) | 1.76 (0.17─18.32) | 0.59 (0.11─3.24) |
| p-STAT3 (geo MFI) | Low | Ref | Ref | Ref |
|  | High | 12.4 (3.76─41.15)*** | 18.0 (1.69─191.5)* | 25.2 (2.48─255.9)** |
| p-STAT3 (%) | Low | Ref | Ref | Ref |
|  | High | 0.53 (0.15─1.83) | 0.42 (0.06─3.15) | 0.35 (0.06─2.11) |
| **Cohort 3** |  |  |  |  |
| Age | Cont. | 1.06 (1.02─1.11)** | 1.0 (0.95─1.06) | 0.95 (0.9─1.01) |
| ARG1 | Low | Ref | Ref | Ref |
|  | High | 0.15 (0.02─1.26) | 0.06 (0.01─0.63)* | 0.24 (0.02─2.54) |
| Urea pH 7.1 | Low | Ref | Ref | Ref |
|  | High | 645.7 38.1─10939)*** | 67.99 (5.35─864.8)** | 238.2 (13.62─4164)*** |
| Urea pH 9.5 | Low | Ref | Ref | Ref |
|  | High | 8.75 (2.19─35.02)** | 4 (0.66─24.3) | 2.75 (0.63─11.97) |

**Abbreviations:** OR, odds ratio, 95% CI, 95% confidence interval. Signiﬁcance levels: <0.001: ***; <0.01: **; <0.05: *

**Supplementary Table 4.** Factors for grade IV glioma patients’ survival (univariate analysis with Cox proportional hazards model).

|  |  | **E/N** | **median (95% CI)** | **HR (95% CI)** | **p-value** |
| --- | --- | --- | --- | --- | --- |
| **Cohort 1** |  |  |  |  |  |
| Age | Cont. | 56/67 | 12.6 (10.6,17.7) | 1.02 (1,1.04) | *0.128* |
| Sex | F | 18/21 | 12 (5.75,17.7) | Ref |  |
|  | M | 38/46 | 14.4 (8.34,20.0) | 0.86 (0.49,1.51) | *0.598* |
| ECOG PS | 0-1 | 32/41 | 18.33 (13.96,22.1) | Ref |  |
|  | 2-3 | 24/26 | 5.96 (4.24,10.6) | 2.9 (1.68,5) | *< 0.001* |
| Surgery | Other | 47/53 | 11.2 (6.37,14.4) | Ref |  |
|  | Radical | 9/14 | 25.9 (11.99,NE) | 0.32 (0.15,0.67) | *0.002* |
| Stupp’s regimen | No | 13/13 | 5.59 (2.27,11.3) | Ref |  |
|  | Yes | 43/54 | 16.03 (11.47,20.4) | 0.25 (0.13,0.49) | *< 0.001* |
| MGMT promoter | No | 28/30 | 11.3 (6.47,13.0) | Ref |  |
| methylation | Yes | 28/37 | 18.0 (10.78,22.8) | 0.52 (0.3,0.89) | *0.016* |
| CD14^+^ | Low | 33/37 | 10.6 (6.18, 16) | Ref |  |
|  | High | 23/30 | 18 (12.22, 28.5) | 0.59 (0.34,1) | *0.051* |
| CD15^+^ | Low | 29/35 | 10.6 (5.88, 17.7) | Ref |  |
|  | High | 27/32 | 14 (11.27, 20.4) | 0.89 (0.52,1.5) | *0.651* |
| MDSC1 | Low | 26/30 | 12.4 (8.34, 20) | Ref |  |
|  | High | 30/37 | 13 (6.47, 18.2) | 1.06 (0.63,1.8) | *0.828* |
| MDSC2 | Low | 14/20 | 21.4 (5.88, 33) | Ref |  |
|  | High | 42/47 | 11.5 (7.75, 14.4) | 2.11 (1.14,3.9) | *0.018* |
| MDSC3 | Low | 23/27 | 11.3 (5.72, 17.6) | Ref |  |
|  | High | 33/40 | 16.4 (10.81, 20) | 0.65 (0.38,1.13) | *0.126* |
| MDSC4 | Low | 43/49 | 11.7 (7.23, 16.7) | Ref |  |
|  | High | 13/18 | 17.6 (11.24, NA) | 0.66 (0.35,1.23) | *0.19* |
| **Cohort 2** |  |  |  |  |  |
| Age | Cont. | 32/45 | 13.04 (7.7,19.9) | 1.04 (1,1.08) | *0.039* |
| Sex | F | 14/18 | 11.1 (4.5,19.9) | Ref |  |
|  | M | 18/27 | 15.2 (5.7,28.5) | 0.68 (0.33,1.4) | *0.291* |
| ECOG PS | 0-1 | 14/25 | 21.6 (15.2,NE) | Ref |  |
|  | 2-3 | 18/20 | 5.7 (2.9,8.7) | 3.79 (1.82,7.88) | *< 0.001* |
| Surgery | Other | 31/39 | 10.6 (5.9,16.0) | Ref |  |
|  | Radical | 1/6 | - | 0.13 (0.02,0.97) | *0.046* |
| Stupp’s regimen | No | 8/9 | 2.7 (1.8,8.2) | Ref |  |
|  | Yes | 24/36 | 15.5 (9.2,22.8) | 0.24 (0.11,0.55) | *< 0.001* |
| MGMT promoter | No | 16/18 | 8.5 (3.1,13.0) | Ref |  |
| methylation | Yes | 16/27 | 21.6 (6.2,NE) | 0.33 (0.16,0.71) | *0.005* |
| CD14^+^/PD-L1^+^ | Low | 19/24 | 9.1 (5.7, 15.2) | Ref |  |
|  | High | 13/21 | 21.6 (4.5, NE) | 0.52 (0.25,1.08) | *0.078* |
| p-STAT3 (geo MFI) | Low | 22/30 | 15.4 (8.7, 21.6) | Ref |  |
|  | High | 10/15 | 6.2 (2.7, NE) | 1.16 (0.54,2.48) | *0.698* |
| p-STAT3 (%) | Low | 11/17 | 16.7 (5.9, NE) | Ref |  |
|  | High | 21/28 | 8.9 (4.2, 14) | 1.68 (0.8,3.5) | *0.168* |
| **Cohort 3** |  |  |  |  |  |
| Age | Cont. | 23/32 | 12.52 (6.47-31.34) | 1.03 (0.99,1.06) | *0.12* |
| Sex | F | 6/10 | 23.03 (0.62-NE) | Ref |  |
|  | M | 17/22 | 9.46 (6.37-28.5) | 1.49 (0.58,3.83) | *0.403* |
| ECOG PS | 0-1 | 11/18 | 28.48 (7.75-NE) | Ref |  |
|  | 2-3 | 12/14 | 8.23 (2.27-13) | 2.69 (1.16,6.23) | *0.021* |
| Surgery | Other | 21/27 | 8.34 (5.88-22.8) | Ref |  |
|  | Radical | 2/5 | - | 0.26 (0.06,1.13) | *0.072* |
| Stupp’s regimen | No | 5/5 | 2.27 (0.62-NE) | Ref |  |
|  | Yes | 18/27 | 19.98 (8.21-33) | 0.1 (0.03,0.31) | *<0.001* |
| MGMT promoter | No | 12/14 | 8.23 (2.63-31.3) | Ref |  |
| methylation | Yes | 11/18 | 21.39 (5.88-NE) | 0.54 (0.23,1.22) | *0.138* |
| ARG1 | Low | 14/17 | 8.34 (4.93-28.5) | Ref |  |
|  | High | 9/15 | 22.80 (5.72-NE) | 0.52 (0.22,1.22) | *0.132* |
| Urea pH 7.1 | Low | 6/8 | 8.23 (0.62-NE) | Ref |  |
|  | High | 17/24 | 18.0 (7.75-33) | 0.64 (0.25,1.62) | *0.344* |
| Urea pH 9.5 | Low | 7/16 | - | Ref |  |
|  | High | 16/16 | 7.98 (5.72-13) | 4.52 (1.78,11.47) | *0.002* |

**Abbreviations:** HR, hazard ratio; 95% CI, 95% confidence interval; E, number of deaths; N, total number of grade IV glioma patients; NE, not estimable.

**Supplementary Table 5.** Characteristics of grade IV glioma patients considered in survival analysis

|  |  |  |  |  |  |  |
| --- | --- | --- | --- | --- | --- | --- |
|  |  |  |  |  |  |  |
|  |  | **Participant characteristics** | | | | |
|  |  |  |  |  |  |  |
|  |  |  |  |  |  |  |
|  |  |  |  |  |  |  |
|  |  | **GIV** |  | **Cohort 1** | **Cohort 2** | **Cohort 3** |
|  |  |  |  |  |  |  |
|  |  |  |  |  |  |  |
| **Total number** |  | 92 | **⇨** | 67 | 45 | 32 |
|  |  |  |  |  |  |  |
|  |  |  |  |  |  |  |
| **Sex** |  |  |  |  |  |  |
| Male (n) |  | 59 |  | 46 | 27 | 22 |
| Female (n) |  | 33 |  | 21 | 18 | 10 |
|  |  |  |  |  |  |  |
| **Median Age** |  | 64 |  | 65 | 66 | 65 |
| Range |  | 27-80 |  | 27-80 | 28-79 | 28-79 |
|  |  |  |  |  |  |  |
| **ECOG PS** |  |  |  |  |  |  |
| 0-1 |  | 59 |  | 41 | 25 | 18 |
| 2-4 |  | 33 |  | 26 | 20 | 14 |
|  |  |  |  |  |  |  |
| **Surgery** |  |  |  |  |  |  |
| Other |  | 74 |  | 53 | 39 | 27 |
| Radical |  | 18 |  | 14 | 6 | 5 |
|  |  |  |  |  |  |  |
| **Stupp’s regimen** |  |  |  |  |  |  |
| No |  | 17 |  | 13 | 9 | 5 |
| Yes |  | 75 |  | 54 | 36 | 27 |
|  |  |  |  |  |  |  |
| **IDH status** |  |  |  |  |  |  |
| WT |  | 88 |  | 64 | 44 | 28 |
| Mutated |  | 4 |  | 3 | 1 | 4 |
|  |  |  |  |  |  |  |
| **MGMT status** |  |  |  |  |  |  |
| Methylated |  | 49 |  | 37 | 27 | 18 |
| Not methylated |  | 43 |  | 30 | 18 | 14 |
|  |  |  |  |  |  |  |
|  |  |  |  |  |  |  |
|  |  |  |  |  |  |  |

**Supplementary Table 6.** Factors for grade IV glioma patients’ survival (univariate analysis with Cox proportional hazards model) stratified for sex.

|  |  | **E/N** | **median (95% CI)** | **HR (95% CI)** | **p-value** | **p-interaction** |
| --- | --- | --- | --- | --- | --- | --- |
| **Sex** | **F** | **18/21** | **12 (5.75,17.7)** |  |  |  |
| MDSC1 | Low | 9/11 | 12.6 (5.75, 33) | Ref |  |  |
|  | High | 9/10 | 11.4 (1.81, 14) | 1.43 (0.6,3.6) | *0.449* | *0.462* |
| MDSC2 | Low | 6/8 | 25.28 (1.81, NA) | Ref |  |  |
|  | High | 12/13 | 7.23 (4.5, 12.6) | 2.57 (0.93,7.14) | *0.070* | *0.592* |
| MDSC3 | Low | 7/9 | 12 (0.624, NA) | Ref |  |  |
|  | High | 11/12 | 12 (4.238, 30.7) | 1.05 (0.96,2.71) | *0.928* | *0.238* |
| MDSC4 | Low | 12/14 | 12.3 (5.59, 19.9) | Ref |  |  |
|  | High | 6/7 | 12 (1.81, 33) | 0.93 (0.34,2.54) | *0.892* | *0.444* |
| **Sex** | **M** | **38/46** | **14.4 (8.34,20.0)** |  |  |  |
| MDSC1 | Low | 17/19 | 12.2 (8.25, 20.7) | Ref |  |  |
|  | High | 21/27 | 17.6 (6.37, 20.4) | 0.93 (0.49,1.8) | *0.828* |  |
| MDSC2 | Low | 8/12 | 21.39 (4.93, NA) | Ref |  |  |
|  | High | 30/34 | 11.98 (8.25, 18) | 1.99 (0.90,4.4) | *0.090* |  |
| MDSC3 | Low | 16/18 | 10.7 (5.027, 18.2) | Ref |  |  |
|  | High | 22/28 | 17.3 (10.809, 22.8) | 0.50 (0.25,1.00) | *0.049* |  |
| MDSC4 | Low | 31/35 | 11.7 (6.47, 18.3) | Ref |  |  |
|  | High | 7/11 | 18.2 (10.78, NA) | 0.53 (0.23,1.20) | *0.128* |  |
